# A comprehensive review on indigenous therapeutic approaches in kidney care using Ayush medicine

**DOI:** 10.3389/fphar.2025.1588424

**Published:** 2026-01-30

**Authors:** Niharika, Rabea Parveen, Bushra Parveen, Shahid Umar, Sayeed Ahmad

**Affiliations:** 1 Department of Botany, School of Chemical and Life Sciences, New Delhi, India; 2 Centre of Excellence in Unani Medicine (Pharmacognosy and Pharmacology), Bioactive Natural Product Laboratory (BNPL), New Delhi, India; 3 Department of Pharmaceutics, School of Pharmaceutical Education and Research, New Delhi, India; 4 Department of Pharmacology, School of Pharmaceutical Education and Research, New Delhi, India

**Keywords:** AYUSH, botanical drugs, chronic kidney disease, nephroprotection, traditional medicine

## Abstract

Chronic kidney disease (CKD) presents a significant global health concern due to its progressive impact on kidney function, and associated complications. This narrative review highlights the role of traditional system of medicine and comprehensive approach to managing chronic kidney disease (CKD) including their molecular mechanisms and therapeutic roles. The interdependence of physical, mental, and spiritual wellbeing is acknowledged by AYUSH, and other indigenous traditions, which emphasize on botanical drugs with proven nephroprotective qualities in addition to dietary changes, lifestyle adjustments, and mind-body therapies. For instance, traditional botanical drugs have shown nephroprotective effects in both preclinical and clinical trials. Yoga activities promote overall wellbeing, which is particularly beneficial for individuals with chronic kidney disease. Our aim is to explore and integrate traditional medicine with modern nephrology practices for enhancing CKD management. However, the review presents the brief importance of this integration and challenges such as the need for robust clinical trials to substantiate safety and efficacy. It also discusses the legal and quality control concerns related to botanical drugs. Overcoming these hurdles is paramount for the successful assimilation of traditional medicine systems with modern healthcare practices. The review also contains the synergistic blend of contemporary nephrology with ancient healing systems like AYUSH offering a compelling approach to CKD treatment and nephroprotection.

## Graphical Abstract

**Figure:**
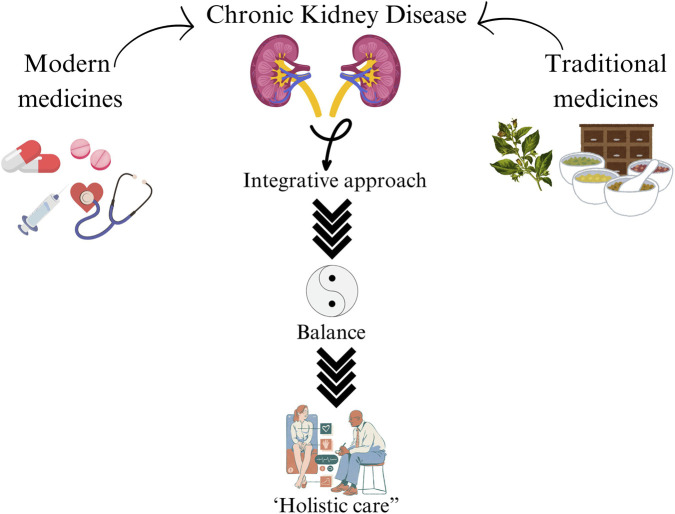

## 1 Introduction

CKD is a global health concern. Even slight deviations in the functioning and structure of the kidneys are known to be associated with increased mortality and risk for multiple organ systems (Mohammadi et al., 2020; Qin et al., 2024). Both acute and chronic kidney failure fall within the potentially fatal category of kidney disease (Kellum et al., 2021). Renal failure is a reduction in glomerular filtration rate (GFR) that affects glomerular elimination functioning (Matas and Rule, 2022).

The kidneys are vital organs in the human body. Endocrine system control, acid-base balance, erythropoiesis, and blood pressure regulation are just a few of the many roles of the kidneys in the body (Imenez Silva and Mohebbi, 2022). CKD is characterized by a GFR of 60 mL/min/1.73 m^2^ resulting from structural or functional abnormalities of the kidneys that persist for over 3 months (dos Santos et al., 2023).

Diagnosis of CKD is done using filtration indicators like cystatin C, albumin, and serum creatinine in urine. Also, measuring the glomerular filtration rate helps in the detection of CKD (Benoit et al., 2020). Kidney abnormalities will affect the regulatory process and alter homeostasis, both of which can be fatal (Garrard and Jones, 2018). It is a critical public health challenge throughout the world. CKD is classified into six successive phases depending upon GFR called the GFR category - Stages 1, 2, 3a and 3b, 4, and 5, and the Albuminuria category, CGA -A1, A2, and A3. Many people with CKD progress to Stage 5 CKD, commonly referred to as end-stage renal disease (ESRD), which is total and irreversible kidney failure (Current Chronic Kidney Disease CKD Nomenclature Used by KDIGO, 2020). CKD frequently develops into ESRD, necessitating kidney replacement *via* dialysis or kidney transplantation (Kalantar-Zadeh et al., 2021). Diabetes and cardiovascular diseases (CVD), persist as the prominent causes of disease and early death in such patients, are directly associated with CKD (Bikbov et al., 2020).

Reducing cardiovascular risk, addressing albuminuria, avoiding nephrotoxins, and modifying medication dosage are all necessary for effectively managing the CKD (Tuttle et al., 2014). There are treatment options available for CKD, such as lifestyle changes, medication, dialysis, and kidney transplantation (Villanego et al., 2020). Making lifestyle changes like quitting smoking, following a balanced diet, exercising regularly, limiting alcohol consumption, and reducing salt intake can significantly slow CKD progression (Luyckx et al., 2017). Medications like ACE inhibitors and ARBs are often prescribed to help control high blood pressure and high cholesterol. A low-protein diet (LPD) is also recommended for patients with CKD. Patients with CKD need to receive routine healthcare services, including cancer screenings and immunizations, just like everyone else (Xie et al., 2016).

Botanical drugs can strengthen and enhance the kidneys and other vital organs (Basist et al., 2022a). Traditional and cultural healing practices among indigenous people utilize an alternative medical system used in AYUSH to manage CKD (Gautam et al., 2021). These practices offer distinct approaches and insights for kidney disease treatment, with an emphasis on holistic approaches (Kalariya et al., 2023). This review discusses the integration of the traditional Indian system of medicine, AYUSH, with modern approaches for the treatment of CKD. The review addresses effective nephroprotective strategies that have been tested and used as per the traditional claims, focusing on botanical drugs, lifestyle modifications, and mind-body therapies. It also focuses on the prospects and challenges in integrating modern medicine with indigenous healing practices while affirming the importance of scientific evidence. There are fundamental aspects, including the need for the development of clinical trials and developing standards for botanical drugs.

## 2 Methodology

### 2.1 Search strategy

**FIGURE 1:**
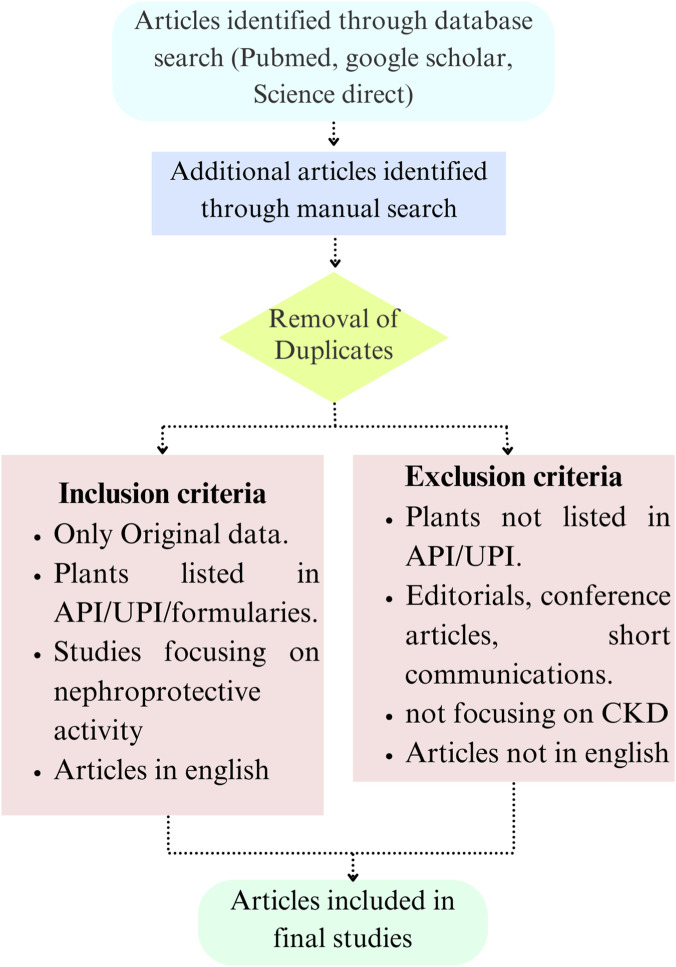
Search strategy used for the study.

Many electronic databases, including Science Direct, Elsevier, Google Scholar, PubMed, Springer, and ACS publications, were used to conduct a thorough literature search to find and compile research assessing the nephroprotective potential of medicinal plants listed in the Unani Pharmacopoeia of India (UPI) and the Ayurvedic Pharmacopoeia of India (API). Including National Formularies of different systems of Ayush. To guarantee that the most current and pertinent data were included in the evaluation, the search was conducted between 2000 and 2025. The search included the following MeSH terms and keyword combinations such as: “nephroprotective plants,” “medicinal plants,” “CKD treatment,” “indigenous healing and ckd”, “modern treatment of CKD,” “phytochemistry CKD,” “CKD mechanism,” and “epidemiology and prevalence of CKD”, AND (“nephroprotective” OR “kidney protective”) AND (“Ayurveda” OR “Unani” OR “traditional medicine” OR “herbal medicine”) AND (“Ayurvedic Pharmacopoeia of India” OR “API” OR “Unani Pharmacopoeia of India” OR “UPI”). Studies that addressed the role of medicinal plants and their therapeutic qualities in the management of CKD were specifically targeted for inclusion. Only English-language studies with original research focusing on *in vitro, in vivo,* or clinical studies, experimentally inducing nephrotoxicity, the use of medicinal plants listed in API, UPI, and the evaluation of the nephroprotective effects of the plants. Studies that lack the mechanistic outcomes and Ayush-based interventions were excluded (Figure 1). All the studies were evaluated according to ConphyMP guidelines (https://ga-online.org/best-practice/#conphymp).

This narrative review gathers and assesses the available data for the treatment of CKD using Indian traditional medicine systems, including Ayurveda, Siddha, Unani, Yoga and Naturopathy, and TCM. Even though a number of studies highlight the advantages of specific therapies, there is not a comprehensive overview that incorporates these into contemporary CKD therapy. The purpose of this review is to close that gap and to direct the future studies towards successful, integrative treatment approaches.

## 3 Results and discussion

### 3.1 Chronic kidney disease (CKD)

CKD is a prevalent global health issue characterized by the irreversible loss of kidney function over the years, often remaining asymptomatic until advanced stages (Palo et al., 2023). It affects approximately >10% of the population worldwide, amounting to more than 800 million people, with greater prevalence in women, older adults, racial minorities, and others with hypertension and diabetes (Kovesdy, 2022; Huang et al., 2023; Simonini and Vezzoli, 2023). Other contributing factors include glomerulonephritis (Hu et al., 2023), autoimmune disease (Rovin, 2020), polycystic kidney disease, lifestyle changes (Bergmann et al., 2018), obesity, and aging (Arabi et al., 2023). There is substantial morbidity and death linked to CKD, following premature death, cardiovascular disease, and ESRD (Huang et al., 2023). This disease imposes substantial health and socio-economic burdens, particularly in low- and middle-income countries (Kovesdy, 2022). CKD progression involves cellular injury, fibrosis, and the loss of functional nephrons, with environmental toxicants like arsenic, cadmium, and mercury exacerbating the condition (Mishra et al., 2022). The renin-angiotensin system (RAS) also plays a role in the mechanism of CKD (Chen et al., 2020). Despite advancements in treatment, the risk of kidney failure and cardiovascular complications remains high, necessitating ongoing research into novel therapeutic strategies (Eckardt et al., 2023). The global burden of CKD is projected to rise, making it the leading cause of death by 2040, underscoring the urgent need for enhanced prevention and treatment efforts worldwide (Kovesdy, 2022).

### 3.2 Epidemiology

Kidney disease affects approximately 195 million women and 800 million to one billion people worldwide. CKD is the sixth-leading cause of death (Fischl, 2019; Vrijlandt et al., 2023). As a result of the massive expense of renal replacement therapy and reduced life expectancy (van Rijn et al., 2020). Ethnicity and social class impact the incidence, prevalence, and development of CKD within nations (Crews et al., 2018).

**FIGURE 2:**
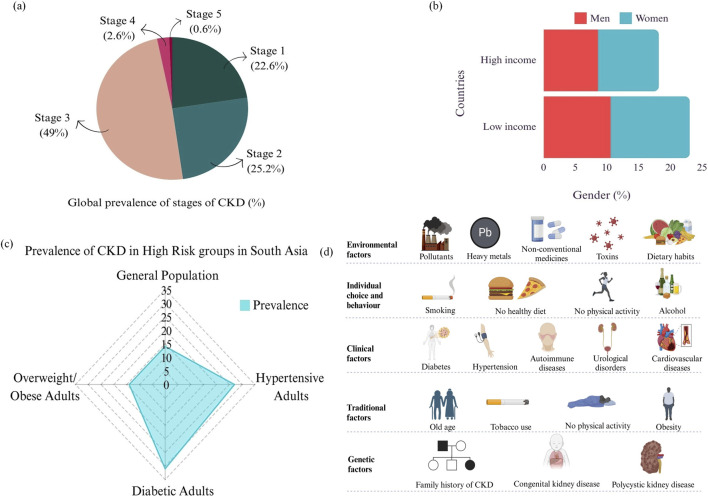
Chronic kidney disease **(a)** Global prevalence **(b)** Prevalence in high-risk countries **(c)** Prevalence in high-risk groups in South Asia (d) risk factors leading to CKD.

According to the recent estimates, CKD affects approximately 10% of the global population. Worldwide, CKD is a major cause of morbidity and mortality. The epidemiology and development of CKD in low- and middle-income nations are poorly understood (Kovesdy, 2022). The Indian Chronic Renal Disease (ICKD) project aims to fill this void by identifying risk factors like cardiovascular disease, renal failure, and progression of CKD in Indian patients (Kumar et al., 2022). A major public health concern in South Asia is CKD, which is exacerbated by a high prevalence of diabetes, hypertension, and lifestyle modifications (Bergmann et al., 2018). A comprehensive review and meta-analysis by (Shrestha et al., 2021) found that the prevalence of CKD was a significant 15.8% in the general population of South Asia, with significantly higher rates across high-risk categories such as those with diabetes or hypertension. Socioeconomic inequality, inadequate awareness, and differences in healthcare access all contribute to this high prevalence and delay in diagnosis and treatment (Figure 2). According to studies, most incidences were in the early stages, providing an essential opportunity for intervention to stop the development of advanced CKD. South Asian countries showed a wide range of prevalence, with Bangladesh and Nepal having especially high percentages (Hasan et al., 2018). Globally, the prevalence of different stages of chronic kidney disease is shown in Figure 2a. About 6%–7% of individuals are in stage 3 CKD, which has the highest prevalence. Conversely, the prevalence rates are lower for the earlier phases (1 and 2) and minimum for the latter stages (4 and 5). According to this distribution, many cases of CKD are discovered in the middle stages, which may present chances for early intervention to stop the disease’s progression. The prevalence of CKD by gender in high- and low-income nations is contrasted in the middle bar chart. With a significant gender difference women exhibit a somewhat greater frequency than males in both economic groups. CKD prevalence seems to be higher in low-income nations (Figure 2b). This demonstrates the gender and socioeconomic differences in the burden of CKD, with low-income groups possibly being at higher risk because of a lack of access to healthcare resources (Stanifer et al., 2018). In comparison to the overall population, the radar graphic on the right shows the prevalence of CKD in South Asia’s high-risk groups, such as individuals with diabetes, hypertension, and overweight/obesity (Figure 2c). Compared to individuals in the highest socioeconomic quartile, those in the lowest have a 60% greater chance of developing advanced CKD. The primary causes of CKD in all high- and middle-income countries and many low-income countries are diabetes and hypertension. Diabetes affects 285 million (6%) adults globally, accounting for 30%–50% of all CKD, and is predicted to rise by 69% in high-income nations and 20% in low- and middle-income countries by 2030 (Chan, 2016; Morton et al., 2016). Developing strong surveillance systems is advised to enhance data on CKD incidence and prevalence, directing initiatives to lower CKD-related morbidity and death (National Center for Chronic Disease Prevention and Health Promotion (U.S.). Division of Diabetes Translation, 2019; Hsu et al., 2021).

The risk factors associated with CKD includes age, hypertension, hepatitis, diabetes, smoking, obesity, and family history of kidney disease (Figure 2d). Other factors such as race, ethnicity, and genetics also play an important role in the progress of CKD. Recent research has also identified environmental factors, such as air pollution and exposure to heavy metals, as potential risk factors for CKD (Pradeepa and Mohan, 2021; Mishra et al., 2022). CKD is associated with an elevation in high risk of CVD, including heart attack and stroke (Olanrewaju et al., 2023). It is also a major risk factor for kidney failure, which requires dialysis or kidney transplantation for treatment. Recent studies have also linked CKD to an increased risk of cognitive impairment and dementia (Blankenship et al., 2024).

### 3.3 Mechanisms responsible for CKD

**FIGURE 3:**
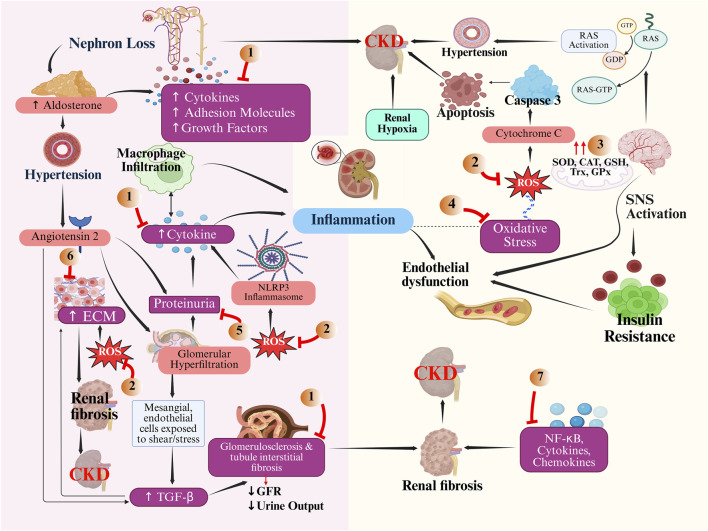
Mechanism showing different metabolites acting on various signals of chronic kidney disease pathway. The image illustrates how various metabolites are responsible for reducing chronic kidney disease in the human body. Metabolites act upon pathways that cause renal fibrosis, proteinuria, oxidative stress, nephron loss, hypertension, *etc.* The numerical in the figure denotes the following Metabolites, 1) *Acacia Senegal* (L.) Britton, *Daucus carota* L., *Crocus sativus* L., *Cucumis melo* L.; 2) *Apium graveolens* L., *Cichorium intybus* L, *Coriandrum sativum* L., *Punica granatum* L., Puerarin, Genkwanin; 3) *Asparagus racemosus Willd*., *Gymnema sylvestre* (Retz.) R. Br. ex Sm.; 4) *Acacia Senegal* (L.) Britton, *Acorus calamus* L., *Aegle marmelos* (L) Correa., *Asparagus racemosus* Willd., *Azadirachta indica* A. Juss. ; 5) *Hibiscus Sabdariff*a L., *Nelumbo nucifera* Gaertn., *Sesamum indicum* L., *Boswellia serrata* Roxb. Ex Colebr. ; 6) *Allium sativum* L.; 7) *Gymnema sylvestre* (Retz.) R. Br. ex Sm.

CKD progression starts from the glomeruli, resulting in higher glomerular capillary hydrostatic pressure and single-nephron glomerular filtration load, triggering glomerular damage and, tangentially, tubular injury (Agarwal and Nath, 2019). Hyperfiltration causes massive endothelial cellular damage by increasing wall stress, which can result in disassociation and podocyte loss, as well as accelerated strain on mesangial cells. It encourages them to generate inflammatory mediators and extracellular matrices, such as transforming growth factor β (TGF-β) or platelet-derived growth factor isoforms (Empitu et al., 2025). The mechanism of CKD is given in Figure 3.

**FIGURE 4:**
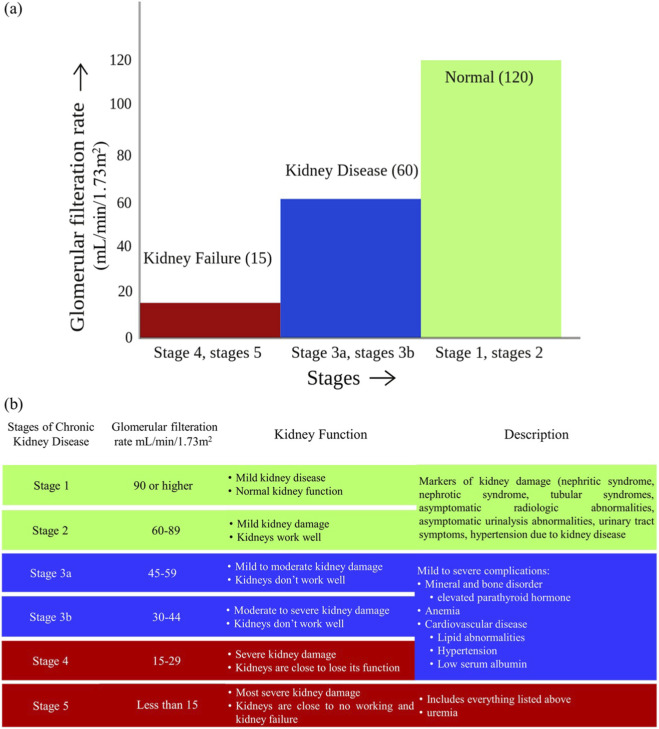
Glomerular filtration rate **(a)** Rate in mL/min/1.73 m^2^ and **(b)** GFR at different stages of chronic kidney disease.

Human nephrons form between weeks ranging 12 to 36 of gestation, with an average of 950,000 nephrons per kidney (tend to range from 200,000 to >2.5 million). After this time, no new nephrons can be produced. To meet increased renal demands, available nephrons grow during development (Chevalier, 2020). In addition, GFR decreases with age. A complete loss of nephrons as well as a consequent reduction in GFR characterize CKD (Figure 4) (Rule et al., 2010). The lowering the number of properly functioning nephrons provokes both cellular and molecular actions which thus promote compensatory development of the remaining units in addition to maintaining renal function (Brenner et al., 2017). These compensatory changes may stimulate mechanisms that result in pathological changes like tubular atrophy and cysts (Stepanova et al., 2024).

Chronic inflammation is caused by many biological signal transduction mechanisms constituting the vasculature and the immune system, stimulating the aggregation of inflammatory cytokines in the tissue (Poveda et al., 2016). Fibrosis is a massively complex cellular repair procedure that takes place in response to injury which results in disruption of this process results in abnormal deposition of extracellular matrix proteins, including collagens. It is exclusively influenced by pro-fibrotic and inflammatory cytokines like the TNF-α, TGF-β, fibroblast growth factor-2, ILs, and platelet-derived growth factor. The extracellular matrix replaces parenchymal tissue in these processes. EMT, induced by various factors such as TGF-β, IL-1β, and angiotensin II, is yet another essential factor for kidney fibrosis (Wu et al., 2022).

It has been reported that CKD patients have relatively high levels of oxidative stress. Reactive Oxidation Species like O^2^•– ion, •OH radical, or H_2_O_2_ are important intracellular messengers; however, too much ROS production can be harmful (Kochi et al., 2018). Higher Ang II levels also add to the excessive production of ROS since Ang II is a potent inducer of NADPH, which is the major source of superoxide. Oxidative stress aggravates inflammation and fibrosis (Kamhieh-Milz et al., 2023).

Renin and ACE alone or in combination with other RAS elements produce angiotensin II. It promotes the growth of the ECM *via* a variety of signal transduction pathways, consequently leading to renal fibrosis. Furthermore, the RAS regulates a variety of pro-fibrotic elements that contribute to the thickening of the ECM, lowering GFR, and causing podocyte death in glomeruli (Qiu et al., 2025).

### 3.4 Challenges with modern system of medicine in the treatment of CKD

The treatment of CKD presents many difficulties for the modern medical system, which hinders the results for patients and healthcare administration (Wang et al., 2022). A full cure is still a breakthrough, particularly for advanced stages, which limit treatment options to dialysis or kidney transplants, both of which are expensive and come with consequences like diabetes and hypertension (Bhandari et al., 2016). Drugs for one problem may make another worse. Electrolyte imbalances and an increased risk of infection are among the side effects that can arise from long-term usage of required medications like diuretics and ACE inhibitors (Fried et al., 2013). CKD is asymptomatic in its early stages, it is often detected late, which makes prevention and successful treatment more difficult (Palo et al., 2023). Another challenge is the current “one-size-fits-all” approach to CKD care since customized care based on lifestyle and genetic factors is still lacking. The efficacy of treatment is impacted by the fact that many patients find it difficult to adhere to rigorous dietary and lifestyle modifications. It is necessary to prioritize early diagnosis, affordable treatments, and individualized, preventive measures to enhance CKD care (Terker et al., 2022).

### 3.5 Indigenous healing approaches for CKD as an alternative

**FIGURE 5:**
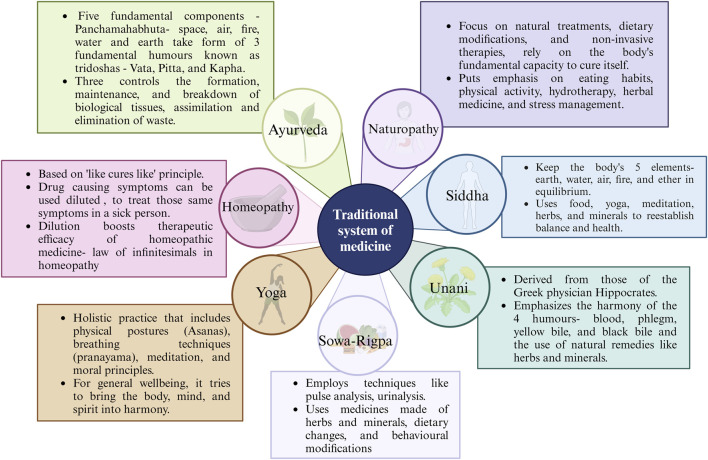
Various classes used in Indian Traditional Indian Medicine.

Cultural safety in healthcare is crucial, as demonstrated by indigenous healing approaches for CKD. Especially while they are undergoing treatment for kidney disease, cultural safety makes sure that indigenous peoples feel valued and secure in the healthcare system (Smith et al., 2021). Botanical drugs and spiritual practices are examples of traditional healing methods that are frequently prioritized in community-centred kidney care (Castelino et al., 2019). The Ayush system of medicine encompasses a rich tapestry of healing practices, including Ayurveda, *Yoga*, *Siddha*, *Unani*, *Homeopathy,* and *Sowa Rigpa*, which have been utilized for centuries and are deeply rooted in the cultural heritage of India to treat a variety of ailments. These systems are deeply rooted in botanical drugs and natural products, documented extensively in ancient texts and modern research (Figure 5) (Javed et al., 2024). For instance, the Ayurvedic Pharmacopoeia of India lists 621 single botanical drugs derived from 393 species, highlighting their diverse therapeutic uses (Yao et al., 2023). The therapeutic potential of these botanical drugs is recognized for their wide range of pharmacological properties (Gaurav et al., 2022). The shift towards traditional medicine from modern medicine is partly due to the perceived safety and affordability of these treatments, although monitoring for contamination is necessary to ensure consumer safety and efficacy of botanical drugs (Gyamfi, 2021).

#### 3.5.1 In *Ayurveda*

The goal of Ayurveda, an age-old South Asian medical system, is to achieve equilibrium both inside the individual and with the surroundings. *Vata*, *pitta*, and *kapha* are the three main doshas that are used to classify people and determine their physiological, psychological, and physical characteristics (Shalini et al., 2021). This categorization, called “*Prakriti*,” directs diagnosis and therapy, enabling extremely individualized care (Jnana et al., 2020). Furthermore, the *Nidan Panchaka* approach, a diagnostic framework consisting of five components *Nidana* (cause), *Purvarupa* (early symptoms), *Rupa* (main symptoms), *Upashaya* (therapeutic tests), and *Samprapti* (pathogenesis) is used in Ayurveda to provide a thorough understanding of the genesis and development of the disease (Ashu and Rakesh Sharma, 2023).

Ayurveda places a strong emphasis on dietary and lifestyle changes as important preventative strategies to preserve *doshic* balance and delay the emergence of chronic illnesses like chronic kidney disease (Chaudhary et al., 2025). Maintaining health and longevity requires a regimen that consists of a *dosha* appropriate balanced diet, exercise, and sleep. By promoting indigenous healing practices in daily habits that complement natural cycles, Ayurvedic principles increase resistance to illness (Asutkar et al., 2023). Several botanical drugs and formulations like *Punarnava*, *Gokshura*, and *Shatavari* are utilized for their nephroprotective properties, which improve urine function and preserve kidney health (Sachdeva et al., 2023).

The goal of Ayurvedic treatment for chronic kidney disease (CKD) is to restore equilibrium using customized therapies, such as *Rasayana Chikitsa* (rejuvenative therapies), *Pachana* (digestive aids), and *Deepana* (digestive stimulants) (Rahul et al., 2025). Botanical drugs with nephroprotective and anti-inflammatory qualities, such as NEERI KFT and *Chandanasava*, are well-known for easing symptoms and enhancing kidney function (Gaurav et al., 2023; Vinothkanna et al., 2023). To improve organ health and eliminate accumulated poisons (*ama*), therapies such as Panchakarma are utilized, particularly *Basti* (medicated enema). Blood purification and diuretic botanical drugs help cleanse the kidneys and enhance urine flow. In addition to treating CKD symptoms, Ayurveda’s holistic methods fortify the body’s defenses against the disease’s advancement (Sharma et al., 2020).

#### 3.5.2 In *Unani*

*Hippocrates* and *Galen* laid the groundwork for Unani medicine, commonly referred to as Greco-Arabic medicine, which was expanded upon by academics such as *Razi* and *Ibn Sina* (Akhtar et al., 2021). It is founded on the equilibrium of the four humors, which affect a temperament of an individual and wellbeing: phlegm, blood, yellow bile, and black bile. Promotive, preventative, curative, and rehabilitative elements of health are all addressed in the Unani holistic approach. The foundation of wellbeing in Unani philosophy is *asbab-e-sittah-zarooriah*, which includes balance of air, food, sleep, mental and physical exercise, excretion, and retention (Lari et al., 2017).

Prevention seeks to preserve humoral balance by adopting dietary and lifestyle habits that are appropriate for a person’s temperament (Kopaei, 2016). To fight against kidney illnesses like CKD, natural nephroprotective botanical drugs are utilized (Akhtar et al., 2021). Moreover, regular treatments like cupping, massage, and venesection are advised to support the body’s detoxification and homeostasis, especially for individuals who are susceptible to kidney illness (Firdaus, 2018).

The Unani system treats renal problems by combining pharmacology, nutrition, botanical remedies, and regimental therapies. Important remedies for diseases such as nephrolithiasis include *Sharbat-e-Bazoori Motadil* and *Qurs-e-Kaknaj*, which have been proven to dissolve kidney stones (Khan et al., 2022; Khan and Tariq, 2021). Numerous Unani formulations, such as *Sharbat-e-Bazoori Motadil*, which contains anti-inflammatory and antioxidant qualities that aid in the fight against nephrotoxicity. While treatments like cupping and diuresis help the body rid itself off toxins, compounds like *Majoon Hajrul Yahood* are employed for their lithotriptic effects (Ajij Ahmed Makbul and Jahan, 2020). Studies on botanical drugs like *Glycyrrhiza glabra* L., Fabaceae and *Apium graveolens* L., Apiaceae have shown encouraging outcomes in their traditional use in treating nephrotoxicity (Naushad et al., 2021; Basist et al., 2022b).

#### 3.5.3 In *yoga* and naturopathy

Yoga is an age-old Indian discipline that combines breathing techniques (pranayama), meditation (*dhyana*), physical postures (*asanas*), and ethical precepts (*yama* and *niyama*) to promote overall health. Yoga stimulates neurohormonal pathways that increase parasympathetic neural activity and decrease sympathetic nervous activity by bringing the mind, body, and spirit into balance (Kauric-Klein, 2022). This promotes emotional stability, enhances mental health, and controls stress, which ultimately helps in the management and prevention of chronic illnesses (Vaishnav et al., 2022), boost cardiovascular health by lowering blood pressure, improving variability in heart rate, and encouraging calm (Silwal et al., 2023).

Chair yoga and guided meditation have been shown in studies to improve the quality of life for hemodialysis patients by lowering blood pressure, anxiety, and depression (Vaishnav et al., 2022). An integrative strategy includes yoga and naturopathy-based lifestyle therapies benefit renal function and quality of life. Furthermore, a 6-month yoga program has been linked to improved renal function and better blood pressure management in CKD patients (Pandey et al., 2017). Through the enhancement of antioxidant enzyme activity and the reduction of lipid peroxidation, yoga activities such as *Hatha yoga* contribute to reducing the impact of oxidative stress in ESRD, improving kidney function overall (Gordon et al., 2013).

#### 3.5.4 In *Siddha*

One of the India’s oldest traditional medical systems, the Siddha medicines seek to promote holistic health by balancing a person’s mental, social, physical, and spiritual wellbeing (Nanda, 2023). It was created by mystical saints called *Siddhars* and has its roots back to the ancient Dravidian civilization (Sieler, 2022). It is founded on the idea of medicinal alchemy, aims to restore damaged organs while preserving equilibrium among the three humors: *pitta, kapha*, and *vata* (Analysis et al., 2018). The 96 *Thathuvams*, which include humors like *Azhal*, *Vali*, and *Iyam* and components like *Panchaboothas* (the five elements of nature), are central to its philosophy (Shanmugam et al., 2024).

Siddha treatment prevent illness by using botanical drugs and formulations, nutritional advice, and lifestyle changes (Parvathy et al., 2023). The Siddha system also promotes yoga, meditation, and regular exercise as ways to keep the body and mind in balance. By avoiding the development of stones and eliminating toxins, preventive botanical infusions such as *Sirupeelai Samoola Kudineer* (SK) enhance the body’s natural healing and detoxification processes while promoting diuresis and kidney health (Veeraraghavan et al., 2015).

Polyherbal formulations with nephroprotective qualities are part of the Siddha approach to kidney health. *Sphaeranthus amaranthoides* extract has demonstrated effectiveness in preventing damage to kidney tubules (Rethinam et al., 2022), whereas *Amirthathi churnam* is utilized to reduce urinary calculi and manage nephrolithiasis symptoms (Nainarpandian, 2018). In addition to preventing kidney stones, *SK* infusion improves antioxidant levels and raises urine pH, both of which support renal health. These treatments improve the patient’s quality of life and total kidney function by preventing more renal injury and promoting kidney recovery through a comprehensive, multifaceted strategy (Veeraraghavan et al., 2015).

#### 3.5.5 In *homeopathy*

An alternative medicine approach developed in 1796 by *Samuel Hahnemann*, homeopathy is based on the idea that “like cures like,” meaning that a substance that produces symptoms in a healthy individual may also alleviate those same symptoms in a sick person. The fundamental principles of homeopathy include the *Law of Similars*, a customized care, and the use of small dosages of remedies to promote the body’s natural healing process (Drofenik, 2019). To maximize their therapeutic potential, homeopathic remedies are generated from both natural and artificial sources, including botanical drugs, minerals, creatures, and manufactured materials, using a process known as *potentization* that involves vigorous shaking and repeated dilutions (Hamre et al., 2023).

Using individualized diluted remedies, to lower oxidative stress, inflammation, and the degradation of lipids in the kidneys, is one of the homeopathy’s preventive strategies. A safe, low-risk preventive method for people who are prone to kidney problems, homeopathic remedies promote overall kidney health by promoting early interventions and modifying their treatment plan as symptoms change (Rasel et al., 2020). Furthermore, homeopathic remedies for CKD are based on customized prescriptions meant to stabilize kidney function and lessen oxidative stress without the need for intrusive procedures (Kalariya et al., 2023). According to studies, homeopathic treatments can successfully lower CKD patients serum creatinine levels, which may postpone the need for dialysis (Rasel et al., 2020). For example, homeopathic medication has been shown to enhance kidney function indicators and decrease tumor size in kidney-related tumors without the need for traditional modern therapies like chemotherapy or surgery (Gaertner et al., 2014). For kidney diseases, homeopathy offers a comprehensive, side-effect-free approach that supports kidney health and overall longevity in life by treating both symptoms and underlying causes (Nambison et al., 2024).

#### 3.5.6 In *Sowa Rigpa*

Tibetan medicine, also known as Sowa Rigpa, is a traditional medical system that dates back approximately 2,000 years to the Himalayan and Central Asian regions (Kloos, 2020). Like the Ayurvedic ideas of *Tridosha* and *Panchamahabhuta*, Sowa Rigpa has its roots in Buddhist philosophy and is founded on the theories of *Nespa gSum* (three humors) and *Jung-wa-lna* (five elements). The method is based on re-establishing the body’s equilibrium through dietary recommendations, lifestyle changes, and natural cures made from a combination of metals, minerals, and botanical drugs (Kloos, 2020). To generate individualized treatments and healing practices that target the underlying causes of health imbalances, these are converted into a variety of forms, including decoctions, powders, pills, pastes, and concentrates (Jawanjal, 2018).

Sowa Rigpa places a strong emphasis on preserving balance between the body’s humors and components to prevent CKD. This is accomplished by following certain dietary recommendations that enhance kidney health in addition to adopting lifestyle choices that encourage indigenous healing, mental and physical equilibrium. Enhancing renal function is the main goal of the holistic approach to combat the variables that contribute to the progression of CKD (Jawanjal, 2018). In fact, to reduce inflammation and oxidative stress, Sowa Rigpa also uses botanical drugs that are well-known for their antioxidant qualities. Sowa Rigpa seeks to avoid kidney disease while promoting general wellbeing by emphasizing early identification and upholding a balanced lifestyle (Kloos, 2020).

Botanical formulations, dietary changes, and lifestyle adjustments are all part of Sowa Rigpa’s CKD treatment plan, which aims to manage renal damage and enhance kidney function. For example, Cordyceps is one remedy that demonstrated promise in lowering urine protein levels and safeguarding renal tubules, particularly in patients with diabetic nephropathy (DN), *via* controlling cell autophagy processes (Pu et al., 2021). Likewise, Siwei Jianghuang Decoction Powder (SWJH), another formulation, has shown promising results in reducing serum creatinine, blood urea nitrogen, and other kidney indicators in diabetic nephropathy models through the modulation of important molecular expressions linked to kidney damage and inflammation (Lai et al., 2018). In addition to supportive lifestyle changes, these natural therapies are intended to reduce renal decline, improve kidney function, and raise patients’ quality of life.

### 3.6 Nephroprotective botanical drugs reported in Ayush

Botanical drugs are an extremely valuable and necessary source of nutrition for humans. Most of the drugs used in Ayush medicines are used to treat CKD due to the presence of metabolites (Gaurav et al., 2022). Botanical drugs are proven to be nephroprotective agents, and they are used to improve renal health and reverse renal damage (Khatoon et al., 2019). Consequently, numerous evidence shows the possible benefits of significant modern-day medications of ancient knowledge of plant therapeutic implements. Botanical drugs can maintain the kidneys and other vital organs while also providing them with strength.

In the Ayush system, several botanical drugs are known to possess nephroprotective properties, meaning they could protect and improve the health of the kidneys. One such plant is *Boerhavia diffusa* L., Nyctaginaceae (Gaurav et al., 2022), which has been traditionally used to treat various kidney-related disorders such as renal calculi, nephritis, and urinary tract infections. *Tribulus terrestris* L., Zygophyllaceae (Sharma et al., 2017), has diuretic and anti-inflammatory properties and helps to improve kidney function. *Curcuma longa* L., Zingiberaceae*,* helps in decreasing bilirubin, creatinine, urea, and total protein, which leads to renal failure (Thuawaini et al., 2019). It also reduces inflammation and oxidative stress. *Asparagus racemosus* Willd., Liliaceae*,* maintains phosphorus levels and also acts as an antioxidant (Yadav and Upasani, 2018). *Asparagus racemosus* Willd., Liliaceae (Roy et al., 2018) is a highly revered herb in Ayurveda, known for its rejuvenating properties and ability to support kidney health. *Phyllanthus niruri* L., Phyllanthaceae (Pahmi and Sidratullah, 2021), *G. glabra* L.*,* Fabaceae (Basist et al., 2022b), and *Azadirachta indica* (Seriana et al., 2021) have also been extensively studied for their nephroprotective properties in Ayurveda. *Crateva nurvala* Buch.-Ham., *Brassicales* (Kaushik et al., 2021) is known to improve kidney function and prevent urinary tract infections. *Berberis aristata* DC., Berberidaceae (Thakur et al., 2017) has anti-inflammatory and antimicrobial properties that can help to reduce kidney inflammation and infections. *Withania somnifera* (L.) Dunal., Solanaceae (Boinepally et al., 2018) has been shown to have a protective effect on the kidneys against oxidative damage and nephrotoxicity. Hence, it has been recognized that a wide range of species of medicinal botanical drugs is utilized as diuretics, and also for burning urination in addition to preventing stone formation in kidneys or nephrolithiasis. In kidney diseases, the roots, seeds, leaves, fruits, pods, gum, bark, and entire plant are beneficial (Khatoon et al., 2019; Akhlaq et al., 2021).

Botanical drugs in Unani have healing properties due to the presence of metabolites. Although the Unani system of medicine does not directly address nephroprotection. Hence, its therapeutic philosophies are based on the notions of organ protection, supplying strength, and regulation of the powers (*Quwa*) at their ideal condition (*Ae’tedal*) (Salma Jabeen et al., 2018). A variety of tonic medications restores it to close to balance if there has been a malfunction. If normalization is not achieved, the condition is treated, or the course is slowed down using medications with specific effects. Therefore, we may state that the traditional Unani system of medicine is distinguished by a defensive framework of delaying the progression of the disease (Naz and Sherani, 2014; Parveen et al., 2020). *Unani* practitioners prepare botanical drugs in various ways, including decoctions, infusions, powders, capsules, tablets, and external applications (Standard Operational Procedures SOPs and Physicochemical Standardisation of a classical Unani Formulation- Qurs Mafasil Jadid, 2021; Yuan et al., 2016). Botanical drugs contain metabolites with medicinal effects, including antioxidant, anti-inflammatory, diuretic, and cytoprotective effects, which help to explain their nephroprotective effects. The whole *Satavar* (*A. racemosus*) plant can be brewed into a decoction as a successful treatment for renal problems (Goyal, 2018). The summary of the traditional medicinal plants used in API and UPI is given in Table 1. The *ConPhyMP* guidelines (https://ga-online.org/best-practice/#conphymp) were followed to structure Table 1.

**TABLE 1:** The table comprises of the preclinical studies conducted on medicinal plants mentioned in API and UPI and their extracts used for CKD against induced toxicity highlighting the major pathways and molecular mechanisms showing Phytotherapeutic effects in alleviating kidney disease.

S. No.	Plant name (reference of traditional claims)	Family	Pharmacopoeial name	Part used	Type of extract	Dose	Screening method	Toxicant (dose)	Pathways	Molecular mechanism	References
1.	*Acacia Senegal* (L.) Britton (UPI Part 1, Vol. VI)	Fabaceae	Samagh-e-arabi	Gum	Aqueous extract	0.75% w/w	Adenine (dose not mentioned)	Wistar Rats	GA ↓ superoxide production, cytokine ↑	Oxidative stress, Inflammatory pathway	Kopaei et al. (2016)
2.	*Acorus calamus* L. (UPI Part I, Vol. V)	Araceae	Vaca	Aerial parts	Ethanolic extract	250–500 mg/kg b.w.	Acetaminophen (750 mg/kg b.w.)	Albino rats	AC ↑ renal SOD, catalase, glutathione, glutathione peroxidase	Oxidative stress	Palani et al. (2010)
3.	*Adhatoda vasica* L. (API part I, Vol. I) (UPI Part I, Vol. VI)	Acanthaceae	Vasa, Arusa	Leaf	Ethanolic extract	500 mg/kg b.w.	Gentamicin (80 mg/kg b.w.)	Wistar rats	↑ body weight ↓ in serum urea, serum creatinine, serum protein	Renal Protective activity, Normalizing kidney markers	Kumar et al. (2013)
4.	*Aegle marmelos* (L) Correa. (UPI Part I, Vol. I)	Rutaceae	Belgiri	Leaf	Ethyl acetate and hydroalcoholic extract	200 mg/kg b.w.	Cisplatin (6 mg/kg b.w.)	Wistar rats	Significantly ↓ MDA, SCr, urea and BUN ↑ GSH and catalase	Oxidative stress pathway	Dwivedi et al. (2017)
5.	*Aerva lanata* (L.) Juss. Ex Schult. (API Part 1, Vol. V)	Amaranthaceae	Pattura	Leaf	Ethanolic extract	150 mg/kg/d b.w.	Cisplatin (150 mg/kg/day for 7 days b.w.)	Albino rats	↑ urea, normalized the creatinine, glutathione, serum albumin, and protein, balanced level of Na and K.	Normalize Kidney markers	(Barkavi and Venkatalakshmi, 2015)
6.	*Allium sativum* L. (UPI Part I, Vol.V)	Amaryllidaceae	Seer	Bulbs	Ethanolic, Aqueous extract	150 and 300 mg/kg + 500 mg/kg b.w.	Cisplatin (5 mg/kg b.w.) + Streptozotocin (45 mg/kg b.w.)	Wistar rats	Normal urea nitrogen level	Normalize Kidney markers	Anusuya et al. (2013), Shiju et al. (2013)
7.	*Alkekengi officinarum* Moench (API Part I, Vol. V)	Solanaceae	Kaknaj	fruit	Hydroalcoholic extract	420 mg/kg and 980 mg/kg b.w.	Cisplatin (7 mg/kg/b.w.)	albino rats	↓ blood urea, SCr, uric acid and TBARS	Normalize Kidney markers	Sabahatullah et al. (2010)
8.	*Aloe vera* (L.) Burm. F. (UPI Part I, Vol. I)	Asphodelaceae	Sibr	Leaf	Ethanolic extract	20 mL/kg + 200, 400 and 600 mg/kg b.w.	Gentamicin (40 mg/kg b.w.) + Diclofenac sodium (50 mg/kg b.w.)	Albino rabbits, Wistar albino rats	Prevented ↑ of serum urea and creatinine levels	Normalize Kidney markers	Iftikhar et al. (2015), Virani et al. (2016)
9.	*Apium graveolens* L. (UPI Part I, Vol. II)	Apiaceae	Tukhm-e-Karafs	Seeds and roots	Aqueous extract	500 and 1,000 mg/kg/b.w.	Cisplatin (5 mg/kg/b.w.)	Albino and Wistar rats	↓ KIM-1, normalize kidney function	Oxidative stress, Inflammatory pathway, Apoptotoc pathway	Naushad et al. (2021)
10.	*Asparagus racemosus* Willd. (UPI Part I, Vol. VI)	Liliaceae	Satawar	Root	Hydroalcoholic extract	100,200,400 mg/kg b.w.	Cisplatin (6 mg/kg b.w.)	Wistar and albino rats	↓ serum BUN level, ↑ SOD level, ↓ MDA level	Oxidative Stress Pathway, Normalize Kidney markers	Yadav and Upasani (2018)
11.	*Azadirachta indica* A. Juss. (UPI Part I, Vol. IV)	Meliaceae	Neem	Leaf	Methanolic extract	500 mg/kg b.w.	Cisplatin (5 mg/kg/b.w.)	Albino and Wistar rats	↑ antioxidant enzymes	Oxidative Stress Pathway	Abdel Moneim et al. (2014)
12.	*Boerhavia diffusa* L. (API Part 1, Vol. I)	Nyctaginaceae	Punarnava	Roots	Roots methanolic extract	50, 150, and 300 mg/kg b.w.	Streptozotocin (60 mg/kg b.w.)	Albino rats	Strong antidiabetic, hypolipidemic impact	Oxidative Stress Pathway	Akhter et al. (2019)
13.	*Boswellia serrata* Roxb. Ex Colebr. (API Part 1, Vol. IV)	Burseraceae	Bunduru	Oleo-gum-resin	Methanol insoluble fraction	350 mg/kg/b.w.	Cadmium chloride (3 mg/kg/b.w.)	Albino rats	↓ serum markers and BUN	Oxidative stress, Anti-inflammatory pathway	Alam et al. (2023)
14.	*Cichorium intybus* L. (UPI Part I, Vol. VI)	Asteraceae	Tukhm-e Kasni	Whole plant	Alcoholic extract	500 mg/kg b.w.	Doxorubicin (15 mg/kg b.w.)	Babb/c mice	Improvement in kidney markers	Oxidative Stress Pathway	Amin et al. (2022)
15.	*Cinnamomum verum* J. Presl (UPI Part I, Vol. I)	Lauraceae	Darchini	Bark	Aqueous extract	300 mg/kg b.w.	Streptozotocin (50 mg/kg b.w.)	Wistar rats	Normalize the serum creatinine and urea	Normalize Kidney markers	Odiase and Om’iniabohs (2017)
16.	*Coriandrum sativum* L. (API Part I, Vol I) (UPI Part I, Vol. I)	Umbelliferae	Dhanyaka	Seeds	Ethyl acetate	200 mg/kg and 400 mg/kg b.w.	Gentamicin (100 mg/kg/b.w.)	Wistar rats	↓ SCr, urea, blood urea nitrogen	Oxidative stress, Normalize kidney markers	Lakhera et al. (2015)
17.	*Crocus sativus* L. (API Part I, Vol. IV) (UPI Part I, Vol. VI)	Iridaceae	Zafran	Stigma	Aqueous extract	40 or 80 mg/kg/day b.w.	Gentamicin (80 mg/kg/d b.w., 5 days, starting from day 6)	Wistar rats	↓ SCr, BUN and renal tissue levels of MDA	Oxidative stress, Anti-inflammatory pathway	Ajami et al. (2010)
18.	*Cucumis melo* L. (UPI Part I, Vol. III)	Cucurbitaceae	Khiyarzah	Seeds	Hydroalcoholic extract	250 and 500 mg/kg/b.w.	Gentamicin (100 mg/kg/b.w.)	Swiss albino mice	↓ total blood urea nitrogen, SCr, urea, uric acid	Normalize Kidney markers, Anti-inflammatory pathway	Saleem et al. (2019)
19.	*Cucumis sativus* (API Part I Vol. V) (UPI Part I, Vol. V)	Cucurbitaceae	Khayar	Seeds	Ethanolic extract	100, 250 and 500 mg/kg/b.w.	Alloxan (150 mg/kg/b.w.)	Wistar Rats	↓ SCr, urea	Normalize Kidney markers	Ofoego et al. (2020)
20.	*Cuminum cyminum* Linn. (API Part I, Vol. I)	Apiaceae	Svetajiraka	Seeds	Aqueous extract	100 and 200 mg/kg	Cisplatin (12 mg/kg b.w.)	Wistar rats	↑ antioxidant enzymes ↓ lipid peroxidation, ↓ serum urea and creatinine level	Normalize Kidney markers, Oxidative stress, Anti-inflammatory pathway	Mahesh et al. (2010)
21.	*Curcuma longa* L. (UPI Part I, Vol. I)	Zingiberaceae	Zard Chob	Rhizome	Aqueous extract	100 and 200 mg/kg b.w.	Isoniazid, rifampicin (50 mg/kg b.w.)	Albino rats	↓ serum ALT, AST, ALP, total bilirubin, creatinine, urea, and total protein	Normalize Kidney markers, Anti-inflammatory pathway	Thuawaini et al. (2019)
22.	*Daucus carota* L. (UPI Part I, Vol. VI)	Umbelliferae	Tukhm-e Gazar	Seed	Ethanolic extract	400 mg/kg/b.w.	Gentamicin (100 mg/kg/b.w.)	Albino wistar rats	↓ BUN, uric acid, and creatinine	Normalize Kidney markers, Anti-inflammatory pathway	Sodimbaku et al. (2016)
23.	*Emblica officianalis* Gaertn. (API Part I, Vol. I) (UPI Part I, Vol. I)	Phyllanthaceae	Amalaki, Amala	Leaf	Hydroalcoholic extract	100 mg/kg, 200 mg/kg and 400 mg/kg/b.w.	Cisplatin (12 mg/kg/b.w.)	Wistar rats	↓ SCr, ↑ antioxidant enzyme activity	Oxidative stress, Anti-inflammatory pathway	Purena et al. (2018)
24.	*Elettaria cardamomum (*L.) Maton (UPI Part I, Vol. I)	Zingiberaceae	Suksmaila, Heel Khurd	Seed oil	na	200 mg/kg b.w.	Paracetamol (500 mg/kg b.w.)	Male Sprague Dawley rats	↑ antioxidant capacity	Oxidative stress pathway	Khattab et al. (2020)
25.	*Foeniculum vulgare* Mill. (API Part I, Vol. I)	Umbelliferae	Badiyan	Seeds	Ethanolic extract	300 and 600 mg/kg/b.w.	CCl_4_ (1.0 mL/kg/b.w.)	Wistar rats	↓ MDA, ↑ antioxidant enzymes activity, improved kidney function	Oxidative stress pathway	Barakat et al. (2023)
26.	*Glycyrrhiza glabra* L. (UPI Part I, Vol. I)	Fabaceae	Asl-us-Soos	Roots	Methanolic extract	31.5, 63, and 126 mg/kg/b.w.	Cisplatin (6 mg/kg b.w.)	HEK-293 Cell lines	Antioxidant, anti-inflammatory, histopathological improvement	Oxidative stress, Anti-inflammatory pathway	Basist et al. (2022a)
27.	*Gymnema sylvestre* (Retz.) R.Br. ex Sm. (API Part I, Vol. V) (UPI Part I, Vol. II)	Apocynaceae	Meṣasṛngi, Gurmar Buti	Leaf	Extract	100 mg/kg b.w.	cisplatin (5 mg/kg b.w.)	Rat	↓ KIM-1, MDA, NF-κB, TNF-α, and apoptosis parameters ↑ SOD and CAT activity	Oxidative stress, Anti-inflammatory pathway	Ibrahim (2024)
28.	*Hibiscus Sabdariffa* L. (API Part I, Vol. III)	Malvaceae	Ambasṭhaki	Leaves	Methanolic extract	150 mg/kg/b.w. and 300 mg/kg/b.w.	Streptozotocin (40 mg/kg b.w.)	Wistar rats	↓ SCr, uric acid and urea level, normalize kidney function, ↑ antioxidant enzymes	Oxidative stress, Anti-inflammatory pathway, Normalize kidney markers	Ajiboye et al. (2024)
29.	*Moringa oleifera* Lam. (UPI Part I, Vol. V)	Moringaceae	Sehjana	Leaves	Alcoholic extract	400 mg/kg/b.w.	Renal ischemia-reperfusion (IR) injury	Wistar rats	↑ antioxidant enzymes	Oxidative stress, Anti-inflammatory pathway	Akinrinde et al. (2020)
30.	*Nelumbo nucifera* Gaertn. (API Part- I, Vol. III)	Nelumbonaceae	Kamala	Roots, leaves, flowers	Ethanolic extract	100 mg/kg b.w.	Gentamicin (100 mg/kg bw.)	Wistar albino rats	↓ Urea, Uric acid, creatinine	Normalize Kidney markers	Srivastava et al. (2014)
31.	*Nigella sativa L.* (UPI Part I, Vol. I)	Ranunculaceae	Kalonji	seed	Aqueous extract	200 mg/kg/b.w.	Thioacetamide (100 mg/kg b.w.)	Wistar Albino rats	Restored antioxidant pathway	Oxidative stress pathway	El-Demerdash et al. (2025)
32.	*Piper cubeba* L. f. (API Part I Vol I) (UPI Part I, Vol. I)	Piperaceae	Kankola	fruits	Powder	810 mg/kg and 1,220 mg/kg b.w.	Gentamycin (80 mg/kg b.w.)	Albino rats	Anti-inflammatory	Oxidative stress, Anti-inflammatory pathway	Ahmad et al. (2012)
33.	*Pueraria tuberosa* (Willd.) DC. (API Part I, Vol. V)	Fabaceae	Vidarikanda	Tubers	Hydroalcoholic extract	30 mg/kg/b.w.	Streptozotocin (55 mg/kg/b.w.)	Albino rats	↓ blood glucose, serum urea and Cr concentration	Normalize Kidney markers, Matrix Metalloproteinase-9 expression	Tripathi et al. (2017)
34.	*Punica granatum* L. (UPI Part I, Vol. II)	Lythraceae	Anar	Leaves	Methanolic extract	100–400 mg/kg/b.w.	Gentamicin (80 mg/kg/b.w.)	Wistar rats + gentamicin	Improve kidney function biomarkers exerted antioxidant activity, and ameliorated histological changes	Oxidative stress pathway	Mestry et al. (2020)
35.	*Sesamum indicum* L. (UPI Part I, Vol. II)	Pedaliaceae	Kunjad Siyah	Seeds	Ethanolic extract	500 mg/kg/b.w.	streptozotocin (65 mg/kg/b.w.)	Albino rats	↓ Serum total protein, albumin and globulin, ↑ blood urea, SCr and uric acid.	Normalize Kidney markers	(Bhuvaneswari and Krishnakumari, 2012)
36.	*Solanum nigrum* L. (UPI Part I, Vol. IV)	Solanaceae	Mako	Fruit	Aqueous extract	1 g/L/b.w.	Streptozotocin (60 mg/kg/b.w.)	Wistar rats	↓ BUN, SCr, NO, MDA and control glucose	Oxidative stress pathway, normalize Kidney markers	Azarkish et al. (2017)
37.	*Terminalia chebula* Retz. (API Part I, Vol. I) (UPI Part I, Vol. I)	Combretaceae	Bibhitaka, Haritaki	Fruit	Hydroalcoholic extract	100 and 200 mg/kg/b.w.	Cisplatin (8 mg/kg/b.w.)	Wistar rats	Antioxidant, anti-inflammatory, modulate apoptotic pathway	Oxidative stress pathway, anti-inflammatory, modulates apoptotic pathway	Kalra et al. (2019)
38.	*Tinospora cordifolia (Willd.) Hook.f. and Thomson* (API Part I, Vol. 1) (UPI Part I, Vol. 1)	Menispermaceae	Guduchi	Root	Aqueous extract	100–400 mg/kg/b.w.	Diclofenac (65 mg/kg/b.w.)	Wistar rats	↑ activity of antioxidant enzymes	Oxidative stress pathway	Gaurav et al. (2022)
39.	*Triticum aestivum* Linn. (UPI Part I, Vol. VI)	Poaceae	Nishasta-e Gandum	wheat juice	Methanol	5 mL juice in 1 mL methanol	20% ethanol (5 g/kg/b.w.)	Albino and Wistar rats	↑ activity of antioxidant enzymes	Oxidative stress pathway	Hebbani et al. (2020)
40.	*Zingiber officinale* Rosc. (API Part I, Vol. I) (UPI Part I, Vol. IV)	Zingiberaceae	Sunthi	Roots	Aqueous extract	50–2000 mg/kg/b.w.	Alloxan (150 mg/kg/b.w.)	Albino rats	Prevented glomerular mesangial matrix deposits and protect nephrons, anti-oxidant	Oxidative stress pathway, Glomerular Mesangial matrix	Irshad et al. (2018)

Abbreviations: API, ayurvedic pharmacopoeia of india; UPI, unani pharmacopoeia of india; ↑, increase; ↓, decrease.

### 3.7 Integration of Ayush with modern medicine for the treatment of CKD

Advancing public health through a multifaceted approach, the integration of Ayush (*Ayurveda, Yoga and Naturopathy, Unani, Siddha, Homeopathy*, and *Sowa Rigpa*) into mainstream healthcare systems aims to harness traditional knowledge in conjunction with modern medical practices. Combining both is vital for the *One Health approach*, which recognizes the interrelatedness of human, animal, and environmental health. Ayush system promote preventive measures, such as daily and seasonal regimens, and the use of rejuvenating botanical drugs. These can significantly impact addressing zoonotic diseases, noncommunicable diseases, antimicrobial resistance, and food safety (Gomes et al., 2023). The National Health Policy of 2017 encourages the integration of allopathic and Ayush care in public facilities, highlighting the necessity of evidence-based practice to guarantee accessibility and cost-effectiveness (Garg and Goyanka, 2023). The holistic approach of combining modern medical techniques with Ayush traditions can address physical, psychological, and lifestyle issues that impact health outcomes. Traditional preparations have shown promising results in improving the kidney health and treating underlying cause of CKD. Furthermore, formulations like *Shatavaryadi Ghana Vati* and *Veertharvayadi Ghana Kwath* have been linked to clinically significant changes in several important renal function indicators (Rathor et al., 2025). In a study, followed by treatment of 100 patients with diabetic nephropathy with ayurvedic treatment, *Niruha basti* of *Punarnavadi kvatha* daily with *Goksuradi guggulu*, *Rasayana churna*, and *Varunadi kvatha* for 1 month, patients were analyzed with reduced serum creatinine, blood urea, urine albumin level (Patel et al., 2011). The different botanical drugs acting on various signals of CKD are given in Figure 3. Ayush therapies are an excellent choice for many individuals who lack easy access to modern medical facilities due to their cost-effectiveness and accessibility (Samal and Dehury, 2019). Integration builds trust and acceptance among healthcare providers and patients.

### 3.8 Limitations and challenges with integrating the Ayush with modern medicine

It is difficult to make direct comparisons and synthesize the evidence between different traditional systems for integration of Ayush system with modern medicine. Because the included studies are very diverse in their design, ranging from *in vitro* tests to *in vivo* animal models to a small number of clinical trials. Ayush system has used plant-derived substances to treat CKD, which has demonstrated great therapeutic efficacy with strong pharmacological properties (Khan et al., 2022). The robustness and dependability of the several findings were limited because of use of non-standardized plant extracts. There was a lack of information on controls and dosages, and preparation methods of extracts, results in non-reproducibility of results. Furthermore, there is still an uncertainty regarding the therapeutic significance of metabolites. There is a lack of proper clinical trials. Also, botanical misidentification is one of the major factors that can be avoided by the use of Pharmacopoeial-grade and taxonomically validated botanicals (Lankasena et al., 2024).

Challenges to the integration process include capacity and resource shortages that restrict access to Ayush medications and chances for fostering connections among biomedical and Ayush professionals. The lack of adequate scientific support for Ayush medications is one of the main issues (Patel et al., 2023). Many Ayush treatments lack rigorous clinical proof, like inadequate randomization, lack of blinding, and insufficient published procedures, etc (Appiah et al., 2018). This lack of scientific validation affects the development of integrated treatment protocols. The known problem of contamination with heavy metals is the biggest obstacle to the safe and widespread adoption of AYUSH in international health systems, especially for CKD patients (Sikder, 2024). According to one investigation, almost one out of every five Ayurvedic drugs purchased included significant levels of lead, mercury, or arsenic (Mikulski et al., 2017).

Another major concern is the lack of reliable quality control and standardization in Ayush products (Katiyar et al., 2023). Climate change, soil conditions, and genetic variations in medicinal plants are some of the factors that affect the raw material variability, thereby affecting the quality of Ayush medications (Zhang et al., 2021). Variability in the raw materials, sophistication of Ayush drug manufacture, and the absence of standardized processes all lead to inconsistent product efficacy and quality (Rathod and Chandak, 2019; Adhikari et al., 2019).

The lack of proper regulations governing manufacturing, sales and marketing and thorough labelling requirements that include information on substances, indications, contraindications, and adverse effects increases the risk of consumer misuse and self-medication. (Patel et al., 2023). There’s a need to strictly adhere to guidelines for quality, safety, and effectiveness (Glasl and Khan, 2018).

## 4 Conclusion and future prospects

CKD is a global health issue affecting more than 800 million people, projected to rise much more by 2040, making it the foremost cause of death (Fischl, 2019; Vrijlandt et al., 2023). Therefore, there is an urgent need for effective prevention and treatment approaches for the management of CKD, as no successful treatment is available in modern medicine.

The review provides a comprehensive overview of the potential role of botanical drugs in the treatment of CKD as per the Ayush. It summarizes the evidence that traditional therapeutic methods can offer beneficial supplementary treatments for managing CKD. This review bridges a significant gap and promotes an integrative strategy that uses the strength of both modern medicine and the Ayush system. Traditional practices offer natural remedies, healthy lifestyle guidance, and holistic healing, while modern medicine provides advanced diagnostics and evidence-based treatments (Ng et al., 2024).

**FIGURE 6:**
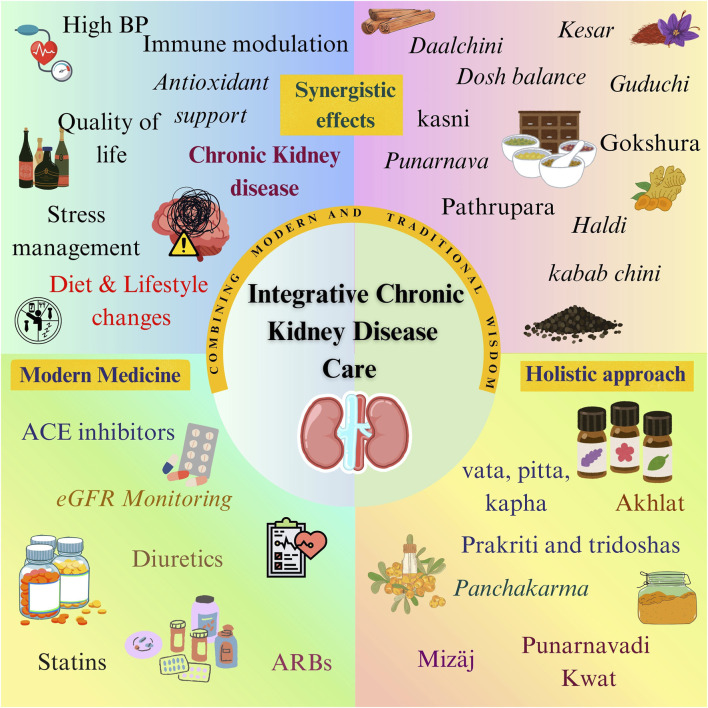
Combining the knowledge of Traditional Indian Medicine with Modern Medicine to improve Kidney health using a patient-centred, holistic approach.

Conducting strong scientific research, including long-term observational and large-scale, multicentred randomized controlled trials, is essential to generate reliable data on the safety, and effectiveness, traditional treatments (Garcia Sanchez et al., 2022). Integrating traditional knowledge with modern science can help create evidence-based therapies and standardized treatment plans. Modern analytical techniques can improve the purity of botanicals (Upton et al., 2020). By setting SoPs for botanicals, keep products consistent across batches and makers. By making regulations for Good Manufacturing Practices and regular quality checks ensures safer, more reliable products, building trust among patients and doctors (Wang et al., 2023). A centralised system should be made to track side effects that can help to detect and maintain safety issues quickly. Scientific validation and research are essential for this integration to bridge the knowledge gap among traditional and modern medicine, guaranteeing the effectiveness and security of traditional treatments (Sebastian, 2024). This combination of Ayush and modern medicine slows down the progression of CKD and enhances the quality of life, minimizing costs and adverse effects (Figure 6).

We believe that this study will lead to better accessible, comprehensive, and efficient care for CKD patients around the world by stimulating interdisciplinary collaboration and more research.
