# Supplementary material for: A comprehensive review on indigenous therapeutic approaches in kidney care using Ayush medicine
Source: Front Pharmacol. 2026 Jan 30;16:1588424. doi: 10.3389/fphar.2025.1588424 (PMC12903127; doi:10.3389/fphar.2025.1588424)
Supplement: Supplementary file 1 [file Table1.docx]

Table 1. The table comprises of the preclinical studies conducted on medicinal plants mentioned in API &UPI and their extracts used for CKD against induced toxicity highlighting the major pathways and molecular mechanisms showing Phytotherapeutic effects in alleviating kidney disease.

| S. No. | Plant name  (Reference of Traditional Claims) | Family | Pharmacopoeial Name | Part used | Type of Extract | Dose | Screening method | Toxicant  (Dose) | Pathways | Molecular Mechanism | Reference |
| --- | --- | --- | --- | --- | --- | --- | --- | --- | --- | --- | --- |
|  | *Acacia Senegal* (L.) Britton  (UPI Part 1, Vol. VI) | Fabaceae | Samagh-e-arabi | Gum | Aqueous extract | 0.75% w/w | Adenine  (dose not mentioned) | Wistar Rats | GA ↓ superoxide production, cytokine ↑ | Oxidative stress, Inflammatory pathway | (Ali et al., 2013) |
|  | *Acorus calamus* L*.*  (UPI Part I, Vol. V) | Araceae | Vaca | Aerial parts | Ethanolic extract | 250-500 mg/kg b.w. | Acetaminophen  (750 mg/kg b.w.) | Albino rats | AC ↑ renal SOD, catalase, glutathione, glutathione peroxidase | Oxidative stress | (Palani et al., 2010) |
|  | *Adhatoda vasica* L.  (API part I, Vol. I)  (UPI Part I, Vol. VI) | Acanthaceae | Vasa, Arusa | Leaf | Ethanolic extract | 500 mg/kg b.w. | Gentamicin (80 mg/kg b.w.) | Wistar rats | ↑ body weight,  ↓ in serum urea, serum creatinine, serum protein | Renal Protective activity, Normalizing kidney markers | (Kumar et al., 2013) |
|  | *Aegle marmelos* (L) Correa*.*  (UPI Part I, Vol. I) | Rutaceae | Belgiri | Leaf | Ethyl acetate & hydroalcoholic extract | 200 mg/kg b.w. | Cisplatin  (6 mg/kg b.w.) | Wistar rats | Signiﬁcantly ↓ MDA, SCr, urea & BUN;  ↑ GSH & catalase | Oxidative stress pathway | (Dwivedi et al., 2017) |
|  | *Aerva lanata* (L.) Juss. Ex Schult.  (API Part 1, Vol. V) | Amaranthaceae | Pattura | Leaf | Ethanolic extract | 150 mg/kg/d b.w. | Cisplatin (150 mg/kg/day for 7 days b.w.) | Albino rats | ↑ urea, normalized the creatinine, glutathione, serum albumin, & protein, balanced level of Na & K. | Normalize Kidney markers | (Barkavi and Venkatalakshmi, 2015) |
|  | *Allium sativum* L.  (UPI Part I, Vol.V) | Amaryllidaceae | Seer | Bulbs | Ethanolic, Aqueous extract | 150 & 300 mg/kg  +  500mg/kg b.w. | Cisplatin  (5 mg/kg b.w.) + Streptozotocin (45 mg/kg b.w.) | Wistar rats | Normal urea nitrogen level | Normalize Kidney markers | (Anusuya et al., 2013; Shiju et al., 2013) |
|  | *Alkekengi* *officinarum* Moench  (API Part I, Vol. V) | Solanaceae | Kaknaj | fruit | Hydroalcoholic extract | 420 mg/kg and 980 mg/kg b.w. | Cisplatin (7 mg/kg/b.w.) | albino rats | ↓ blood urea, SCr, uric acid & TBARS | Normalize Kidney markers | (Sabahatullah et al., 2010) |
| 1. 7. | *Aloe vera* (L.) Burm. F.  (UPI Part I, Vol. I) | Asphodelaceae | Sibr | Leaf | Ethanolic extract | 20 ml/kg  +  200, 400 & 600 mg/kg b.w. | Gentamicin (40 mg/kg b.w.) + Diclofenac sodium (50 mg/kg b.w.) | Albino rabbits, Wistar albino rats | Prevented ↑ of serum urea & creatinine levels | Normalize Kidney markers | (Iftikhar et al., 2015; Virani et al., 2016) |
|  | *Apium graveolens* L.  (UPI Part I, Vol. II) | Apiaceae | Tukhm-e-Karafs | Seeds & roots | Aqueous extract | 500 and 1000 mg/kg/b.w. | Cisplatin  (5 mg/kg/b.w.) | Albino & Wistar rats | ↓ KIM-1, normalize kidney function | Oxidative stress, Inflammatory pathway, Apoptotoc pathway | (Naushad et al., 2021) |
|  | *Asparagus racemosus* Willd.  (UPI Part I, Vol. VI) | Liliaceae | Satawar | Root | Hydroalcoholic extract | 100,200,400 mg/kg b.w. | Cisplatin  (6 mg/kg b.w.) | Wistar & albino rats | ↓ serum BUN level, ↑ SOD level, ↓ MDA level | Oxidative Stress Pathway, Normalize Kidney markers | (Yadav and Upasani, 2018) |
|  | *Azadirachta indica* A. Juss.  (UPI Part I, Vol. IV) | Meliaceae | Neem | Leaf | Methanolic extract | 500 mg/kg b.w. | Cisplatin  (5 mg/kg/b.w.) | Albino & Wistar rats | ↑ antioxidant enzymes | Oxidative Stress Pathway | (Moneim et al., 2014) |
|  | *Boerhavia*  *diffusa* L.  (API Part 1, Vol. I) | Nyctaginaceae | Punarnava | Roots | Roots methanolic extract | 50, 150, and 300 mg/kg b.w. | Streptozotocin  (60 mg/kg b.w.) | Albino rats | Strong antidiabetic,  hypolipidemic impact | Oxidative Stress Pathway | (Akhter et al., 2019) |
|  | *Boswellia serrata* Roxb. Ex Colebr.  (API Part 1, Vol. IV) | Burseraceae | Bunduru | Oleo-gum-resin | Methanol insoluble fraction | 350 mg/kg/b.w. | Cadmium chloride  (3 mg/kg/b.w.) | Albino rats | ↓ serum markers & BUN | Oxidative stress, Anti-inflammatory pathway | (Alam et al., 2023) |
|  | *Cichorium intybus* L*.*  (UPI Part I, Vol. VI) | Asteraceae | Tukhm-e Kasni | Whole plant | Alcoholic extract | 500 mg/kg b.w. | Doxorubicin  (15 mg/kg b.w.) | Babb/c mice | Improvement in kidney markers | Oxidative Stress Pathway | (Amin et al., 2022) |
|  | *Cinnamomum verum* J. Presl  (UPI Part I, Vol. I) | Lauraceae | Darchini | Bark | Aqueous extract | 300 mg/kg b.w. | Streptozotocin  (50 mg/kg b.w.) | Wistar rats | Normalize the serum creatinine & urea | Normalize Kidney markers | (Odiase and Om’iniabohs, 2017) |
|  | *Coriandrum sativum* L.  (API Part I, Vol I)  (UPI Part I, Vol. I) | Umbelliferae | Dhanyaka | Seeds | Ethyl acetate | 200 mg/kg & 400 mg/kg b.w. | Gentamicin  (100 mg/kg/b.w.) | Wistar rats | ↓ SCr, urea, blood urea nitrogen | Oxidative stress, Normalize kidney markers | (Lakhera et al., 2015) |
|  | *Crocus sativus* L.  (API Part I, Vol. IV)  (UPI Part I, Vol. VI) | Iridaceae | Zafran | Stigma | Aqueous extract | 40 or 80 mg/kg/day b.w. | Gentamicin (80 mg/kg/d b.w., 5 days, starting from day 6) | Wistar rats | ↓ SCr, BUN & renal tissue levels of MDA | Oxidative stress, Anti-inflammatory pathway | (Ajami et al., 2010) |
|  | *Cucumis melo* L.  (UPI Part I, Vol. III) | Cucurbitaceae | Khiyarzah | Seeds | Hydroalcoholic extract | 250 & 500 mg/kg/b.w. | Gentamicin (100 mg/kg/b.w.) | Swiss albino mice | ↓ total blood urea nitrogen, SCr, urea, uric acid | Normalize Kidney markers, Anti-inflammatory pathway | (Saleem et al., 2019) |
|  | *Cucumis sativus*  (API Part I Vol. V)  (UPI Part I, Vol. V) | Cucurbitaceae | Khayar | Seeds | Ethanolic extract | 100, 250 & 500 mg/kg/b.w. | Alloxan  (150 mg/kg/b.w.) | Wistar Rats | ↓ SCr, urea | Normalize Kidney markers | (Ofoego et al., 2019) |
|  | *Cuminum cyminum* Linn.  (API Part I, Vol. I) | Apiaceae | Svetajiraka | Seeds | Aqueous extract | 100 & 200 mg/kg | Cisplatin  (12 mg/kg b.w.) | Wistar rats | ↑ antioxidant enzymes,  ↓ lipid peroxidation, ↓ serum urea & creatinine level | Normalize Kidney markers, Oxidative stress, Anti-inflammatory pathway | (Mahesh et al., 2010) |
|  | *Curcuma longa* L.  (UPI Part I, Vol. I) | Zingiberaceae | Zard Chob | Rhizome | Aqueous extract | 100 and 200 mg/kg b.w. | Isoniazid,  rifampicin  (50 mg/kg b.w.) | Albino rats | ↓ serum ALT, AST, ALP, total bilirubin, creatinine, urea, & total protein | Normalize Kidney markers, Anti-inflammatory pathway | (Thuawaini *et al*., 2019) |
| 1. i | *Daucus carota* L.  (UPI Part I, Vol. VI) | Umbelliferae | Tukhm-e Gazar | Seed | Ethanolic extract | 400 mg/kg/b.w. | Gentamicin  (100 mg/kg/b.w.) | Albino wistar rats | ↓ BUN, uric acid, and creatinine | Normalize Kidney markers, Anti-inflammatory pathway | (Sodimbaku et al., 2016) |
|  | *Emblica officianalis* Gaertn.  (API Part I, Vol. I)  (UPI Part I, Vol. I) | Phyllanthaceae | Amalaki, Amala | Leaf | Hydroalcoholic extract | 100 mg/kg, 200  mg/kg and 400  mg/kg/b.w. | Cisplatin  (12 mg/kg/b.w.) | Wistar rats | ↓ SCr, ↑ antioxidant enzyme activity | Oxidative stress, Anti-inflammatory pathway | (Purena et al., 2018) |
|  | *Elettaria cardamomum (*L.) Maton  (UPI Part I, Vol. I) | Zingiberaceae | Suksmaila , Heel Khurd | Seed oil | na | 200 mg/kg b.w. | Paracetamol (500 mg/kg b.w.) | Male Sprague Dawley rats | ↑ antioxidant capacity | Oxidative stress pathway | (Khattab et al., 2020) |
|  | *Foeniculum vulgare* Mill.  (API Part I, Vol. I) | Umbelliferae | Badiyan | Seeds | Ethanolic extract | 300 and 600 mg/kg/b.w. | CCl_4_  (1.0 mL/ kg/b.w.) | Wistar rats | ↓ MDA, ↑ antioxidant enzymes activity, improved kidney function | Oxidative stress pathway | (Barakat et al., 2023) |
|  | *Glycyrrhiza glabra* L.  (UPI Part I, Vol. I) | Fabaceae | Asl-us-Soos | Roots | Methanolic extract | 31.5, 63, & 126 mg/kg/b.w. | Cisplatin (6 mg/kg b.w.) | HEK-293 Cell lines | Antioxidant, anti-inflammatory, histopathological improvement | Oxidative stress, Anti-inflammatory pathway | (Basist et al., 2022) |
|  | *Gymnema sylvestre* (Retz.) R.Br. ex Sm.  (API Part I, Vol. V)  (UPI Part I, Vol. II) | Apocynaceae | Meṣasṛngi, Gurmar Buti | Leaf | Extract | 100 mg/kg b.w. | cisplatin  (5 mg/kg b.w.) | Rat | ↓ KIM-1, MDA, NF-κB, TNF-α, & apoptosis parameters,  ↑ SOD & CAT activity | Oxidative stress, Anti-inflammatory pathway | (Ibrahim, 2024) |
|  | *Hibiscus Sabdariffa* L.  (API Part I, Vol. III) | Malvaceae | Ambasṭhaki | Leaves | Methanolic extract | 150 mg/kg/b.w. & 300mg/kg/b.w. | Streptozotocin (40 mg/kg b.w.) | Wistar rats | ↓ SCr, uric acid & urea level, normalize kidney function, ↑ antioxidant enzymes | Oxidative stress, Anti-inflammatory pathway, Normalize kidney markers | (Ajiboye et al., 2024) |
|  | *Moringa oleifera* Lam.  (UPI Part I, Vol. V) | Moringaceae | Sehjana | Leaves | Alcoholic extract | 400 mg/kg/b.w. | Renal ischemia-reperfusion (IR) injury | Wistar rats | ↑ antioxidant  enzymes | Oxidative stress, Anti-inflammatory pathway, | (Akinrinde et al., 2020) |
|  | *Nelumbo nucifera* Gaertn.  (API Part- I, Vol. III) | Nelumbonaceae | Kamala | Roots, leaves, flowers | Ethanolic extract | 100 mg/kg b.w. | Gentamicin (100 mg/kg bw.) | Wistar albino rats | ↓ Urea, Uric acid, creatinine | Normalize Kidney markers | (Srivastava et al., 2014) |
|  | *Nigella sativa L.* (UPI Part I, Vol. I) | Ranunculaceae | Kalonji | seed | Aqueous extract | 200 mg/kg/b.w. | Thioacetamide  (100 mg/kg b.w.) | Wistar Albino rats | Restored antioxidant pathway | Oxidative stress pathway | (El-Demerdash et al., 2025) |
|  | *Piper cubeba* L. f.  (API Part I Vol I)  (UPI Part I, Vol. I) | Piperaceae | Kankola | fruits | Powder | 810 mg/kg & 1220 mg/kg b.w. | Gentamycin (80 mg/kg b.w.) | Albino rats | Anti-inflammatory | Oxidative stress, Anti-inflammatory pathway, | (Ahmad et al., 2012) |
|  | *Pueraria tuberosa* (Willd.) DC.  (API Part I, Vol. V) | Fabaceae | Vidarikanda | Tubers | Hydroalcoholic extract | 30 mg/kg/b.w. | Streptozotocin  (55 mg/kg/b.w.) | Albino rats | ↓ blood glucose, serum urea & Cr concentration, | Normalize Kidney markers, Matrix Metalloproteinase-9 expression | (Tripathi et al., 2017) |
|  | *Punica granatum* L.  (UPI Part I, Vol. II) | Lythraceae | Anar | Leaves | Methanolic extract | 100-400 mg/kg/b.w. | Gentamicin (80 mg/kg/b.w.) | Wistar rats + gentamicin | Improve kidney  function  biomarkers,  exerted antioxidant  activity, &  ameliorated histological changes | Oxidative stress pathway | (Mestry et al., 2020) |
|  | *Sesamum indicum* L.  (UPI Part I, Vol. II) | Pedaliaceae | Kunjad Siyah | Seeds | Ethanolic extract | 500 mg/kg/b.w. | streptozotocin  (65 mg/kg/b.w.) | Albino rats | ↓ Serum total protein, albumin and globulin, ↑ blood urea, SCr & uric acid. | Normalize Kidney markers | (Bhuvaneswari and Krishnakumari, 2012) |
|  | *Solanum nigrum* L.  (UPI Part I, Vol. IV) | Solanaceae | Mako | Fruit | Aqueous extract | 1 g/L/b.w. | Streptozotocin (60 mg/kg/b.w.) | Wistar rats | ↓ BUN, SCr, NO, MDA & control glucose | Oxidative stress pathway, normalize Kidney markers | (Azarkish et al., 2017) |
|  | *Terminalia chebula* Retz.  (API Part I, Vol. I)  (UPI Part I, Vol. I) | Combretaceae | Bibhitaka, Haritaki | Fruit | Hydroalcoholic extract | 100 and 200 mg/kg/b.w. | Cisplatin (8 mg/kg/b.w.) | Wistar rats | Antioxidant, anti-inflammatory, modulate apoptotic pathway | Oxidative stress pathway, anti-inflammatory, modulates apoptotic pathway | (Kalra et al., 2019) |
|  | *Tinospora cordifolia (Willd.) Hook.f. & Thomson*  (API Part I, Vol. 1)  (UPI Part I, Vol. 1) | Menispermaceae | Guduchi | Root | Aqueous extract | 100-400 mg/kg/b.w. | Diclofenac  (65 mg/kg/b.w.) | Wistar rats | ↑ activity of antioxidant enzymes | Oxidative stress pathway | (Gaurav et al., 2022) |
|  | *Triticum aestivum* Linn.  (UPI Part I, Vol. VI) | Poaceae | Nishasta-e Gandum | wheat juice | Methanol | 5 ml juice in 1 ml methanol | 20% ethanol (5 g/kg/b.w.) | Albino & Wistar rats | ↑ activity of antioxidant enzymes | Oxidative stress pathway | (Hebbani et al., 2020) |
|  | *Zingiber ofﬁcinale* Rosc.  (API Part I, Vol. I)  (UPI Part I, Vol. IV) | Zingiberaceae | Sunthi | Roots | Aqueous extract | 50-2000 mg/kg/b.w. | Alloxan  (150 mg/kg/b.w.) | Albino rats | Prevented glomerular mesangial matrix deposits & protect nephrons, anti-oxidant | Oxidative stress pathway, Glomerular Mesangial matrix | (Irshad et al., 2018) |

*API: Ayurvedic Pharmacopoeia of India, UPI: Unani Pharmacopoeia of India, ↑- increase,* ↓- *decrease*
